# Morphology extraction of fetal ECG using temporal CNN-based nonlinear adaptive noise cancelling

**DOI:** 10.1371/journal.pone.0278917

**Published:** 2022-12-15

**Authors:** Shi Cao, Hui Xiao, Gao Gong, Weiyang Fang, Chaomin Chen

**Affiliations:** School of Biomedical Engineering, Southern Medical University, Guangzhou, China; Vellore Institute of Technology: VIT University, INDIA

## Abstract

**Objectives:**

Noninvasive fetal electrocardiography (FECG) offers many advantages over alternative fetal monitoring techniques in evaluating fetal health conditions. However, it is difficult to extract a clean FECG signal with morphological features from an abdominal ECG recorded at the maternal abdomen; the signal is usually contaminated by the maternal ECG and various noises. The aim of the work is to extract an FECG signal that preserves the morphological features from the mother’s abdominal ECG recording, which allows for accurately estimating the fetal heart rate (FHR) and analyzing the waveforms of the fetal ECG.

**Methods:**

We propose a novel nonlinear adaptive noise cancelling framework (ANC) based on a temporal convolutional neural network (CNN) to effectively extract fetal ECG signals from mothers’ abdominal ECG recordings. The proposed framework consists of a two-stage network, using the ANC architecture; one network is for the maternal ECG component elimination and the other is for the residual noise component removal of the extracted fetal ECG signal. Then, JADE (one of the blind source separation algorithms) is applied as a postprocessing step to produce a clean fetal ECG signal.

**Results:**

Synthetic ECG data (FECGSYNDB) and clinical ECG data (NIFECGDB, PCDB) are used to evaluate the extraction performance of the proposed framework. The statistical and visual results demonstrate that our method outperforms the other state-of-the-art algorithms in the literature. Specifically, on the FECGSYNDB, the mean squared error (MSE), signal-to-noise ratio (SNR), correlation coefficient (R) and *F*_1_-score of our method are 0.16, 7.94, 0.95 and 98.89%, respectively. The *F*_1_-score on the NIFECGDB reaches 98.62%. The value of the *F*_1_-score on the PCDB is 98.62%.

**Conclusion:**

As opposed to the existing algorithms being restricted to fetal QRS complex detection, the proposed framework can preserve the morphological features of the extracted fetal ECG signal well, which could support medical diagnoses based on the morphology of the fetal ECG signal.

## Introduction

Intrauterine hypoxia and congenital heart defects (CHDs) are the main reasons for fetal growth retardation and even death, which is a major risk to the fetus [1]. Therefore, effective fetal monitoring is essential throughout pregnancy to ensure the safety of the fetus and the mother [2]. Continuous fetal heart rate monitoring, i.e., cardiotocography (CTG), has been the most widely used technique for fetal monitoring since the 1960s, with Doppler ultrasound (US)-based fetal heart rate (FHR) monitoring being the most popular [3]. However, fetal monitoring has problematic reliability and does not provide the beat-to-beat fetal HR information that is required for a reliable analysis of the heart rate variabilities [4]. In contrast, the fetal electrocardiogram (FECG) allows for the screening of fetal well-being through the analysis of both the FHR and the fetal ECG waveform morphological features [5]. For example, fetal hypoxia that causes birth defects can be diagnosed by monitoring FHR estimated by fetal QRS complex detection, and the morphological features of the FECG can provide further information to enable the early diagnosis of congenital heart defects (CHDs) [6, 7]. Hence, it would be of interest to extract FECG while preserving the diagnostic morphological information. The FECG signal is extracted from the abdominal electrocardiogram (AECG) recording acquired by surface electrodes that are placed onto the maternal abdomen [8]. Unfortunately, it is a challenge to extract a FECG signal with clean morphology from an AECG recording. The main reason is that in the AECG recording, the FECG component is much weaker than the maternal ECG (MECG) component, and they usually overlap both in the time and frequency domains. As a result, extracting the FECG from an abdominal mixed signal is susceptible to influence from the strong MECG component [9]. Moreover, the AECG recording is usually distorted by various noises, such as maternal electromyogram (EMG), power line interference, baseline drift and white noise [10]. The above interferences would severely disrupt the extraction of the FHR and FECG morphology.

Over the past decades, many methods have been proposed for extracting FECG from the AECG recordings [11]. They can be categorized by filtering techniques, template subtraction (TS) and blind source separation (BSS). The popular filtering techniques include adaptive filtering [7, 12], Kalman filtering [13] and wavelet transforms [14]. Classical adaptive filtering requires a maternal thoracic ECG signal as a reference to remove the maternal ECG component from the abdominal ECG. However, the relationship between the thoracic MECG and abdominal MECG is complexly nonlinear, and the mapping ability of classical adaptive filtering is very limited. TS [15] involves subtracting a synthetic MECG generated by detecting maternal QRS accurately, leaving a residual signal containing the FECG. The main challenge of TS is that the synthetic MECG may not hold the morphology of the maternal ECG component in the abdominal ECG. This drawback degrades the effectiveness of the fetal ECG extraction. The BSS methods include a principal component analysis (PCA) [16], independent component analysis (ICA) [17] and joint approximate diagonalization of eigen matrices (JADE) [18]. The BSS techniques require multiple channels of AECG recordings, which would increase the discomfort for pregnant women. The other drawback of BSS is the inability to directly separate the weak fetal ECG component from the abdominal ECG [19].

Recently, with the development of deep neural networks (DNNs), many researchers have tried to extract FECG signals using the DNN technique [20]. Zhong et al. [21] proposed a deep convolutional encoder-decoder network to directly extract the FECG from the AECG signal. Using a two-dimensional DNN, Xu et al. [22] took advantage of the structured information in the time-frequency domain of the AECG signal to detect fetal QRS complexes without eliminating the MECG component. The model developed by Lee et al. [23] had a deeper architecture, which led to performance improvements. Vo k et al. [3] designed an end-to-end deep neural network to detect fetal QRS complexes from multichannel abdominal ECG recordings. However, there are several deficiencies in the existing DNN-based research: (1) It is difficult to train a model that directly extracts the weak FECG information from the abdominal mixed signal since the MECG usually has a greater amplitude than that of the FECG. A fetal scalp ECG is required for supervised learning of the model, and the scarcity of training data may seriously degrade the generalization performance of the model. (2) An FECG signal extracted by the existing models is still contaminated by residual maternal ECG and noise, resulting in a low signal-to-noise ratio (SNR). Usually, the QRS complexes of the FECG signal extracted by the existing methods can be detected without further processing of the FECG due to the high amplitude of the R peak [24]. However, the detection of smaller waves, such as the P and T waves, would be a challenging task in actual clinical practice. (3) In addition, most research does not consider the particularity of the sequence modeling. For the prediction of a long-term ECG signal, the predicted value *y*_*t*_ at time *t* depends on the state of the long historical information that has been previously observed: {*x*_1_,…,*x*_*t*_}. Therefore, it is not suitable to simply replace the 2D CNN that processes images with a 1D CNN to process signals where the small convolutional kernel cannot perceive the long quantity of historical information within the limited depth of the network.

**Fig 1:**
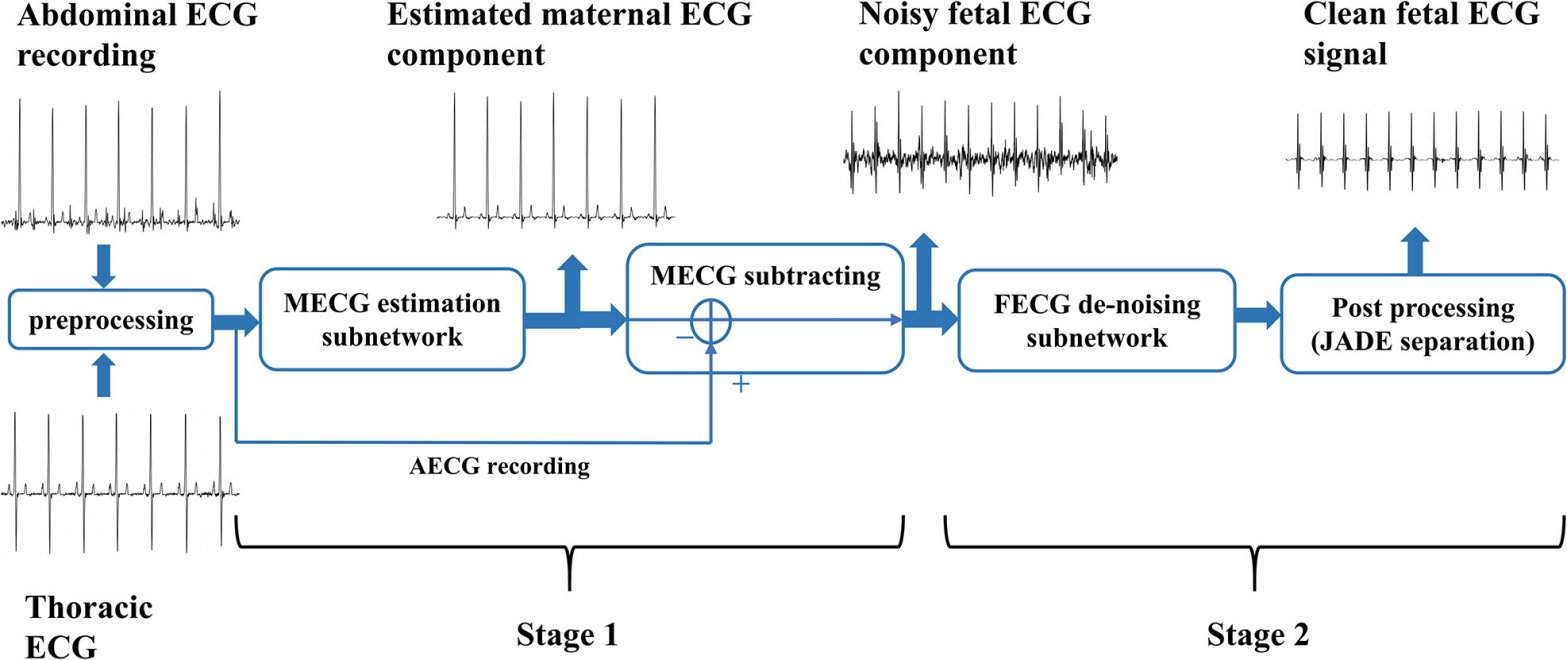
The overall process of the proposed framework. The MECG component is eliminated in stage 1 and the extracted FECG is denoised in stage 2.

In this paper, referring to the architecture of adaptive noise cancelling (ANC), we propose a DNN-based framework to extract the FECG signal from the AECG recording. As shown in Fig 1, the framework consists of two temporal convolutional encoder-decoder networks with residual and skip connections. The first network uses an additional reference signal correlated to the maternal ECG (i.e., thoracic ECG of the mother) to estimate the maternal ECG component in the abdominal ECG. After that, the estimation is subtracted from the abdominal ECG, leaving a residual signal containing the fetal ECG. Then, the second network and a postprocessing (JADE) step remove the residual maternal ECG and noise from the noisy fetal ECG signal to produce a clean fetal ECG signal. Our main research contributions are summarized as follows: (1) We show that using two networks (one for MECG elimination and the other for FECG denoising) is capable of extracting the FECG from the AECG recording with high accuracy for QRS detection while better preserving most of the morphological features, as opposed to using one network model for direct FECG extraction. (2) The proposed method is a combination of the convolutional neural network (CNN) and adaptive noise cancelling (ANC). ANC is capable of adjusting its parameters autonomously to remove the uncorrelated noise components. In the ANC architecture, our model utilizes the error between the current reference signal and the signal to be processed for online optimization and prediction. As a result, our model requires no significant model training or testing while achieving a higher, or comparable performance, to the state-of-the-art methods. (3) For the problem that in the prediction of the long-term ECG signal, the small kernel of the regular CNN cannot perceive the long quantities of historical information within the limited network depth, we use dilated convolution [25] to increase the receptive field and perceive the long quantity of historical information of the ECG signal, which corresponds to the long-term memory of the LSTM. Another benefit of dilated convolution operations is the reduction in computational cost and memory footprint, enabling implementation on resource-constrained devices. In contrast with the recurrent neural network (RNN), our convolution-based model implements the massively parallel computing of the long-term ECG signal via GPU computation and, as such, is rather generally practical.

The rest of this paper is organized as follows. Section 2 presents the experimental data and our proposed model architecture, as well as provides detailed theoretical analyses. In Section 3, experimental results are discussed along with the corresponding interpretations. Finally, the discussion and the conclusion are drawn in Section 4 and Section 5, respectively.

## Materials and methods

### Experimental data

In this work, three available datasets from the FECG synthetic database (FECGSYNDB) [26], Physionet noninvasive fetal ECG database (PNIFECGDB) [27] and set A of Physionet/computing in the cardiology challenge database (PCDB) [28] are used.

The FECGSYNDB is a large database of simulated noninvasive fetal ECG signals, which provides a robust resource that enables reproducible research in the field. The recording obtains two thoracic ECG signals and 32 abdominal mixed signals by treating each abdominal signal component (FECG/MECG or noise signals) as an individual source. The combination of four channels (1, 11, 22 and 32) [28] is used for the evaluation of our algorithm, where the noise level is 0 dB, as suggested by Andreotti et al. [29]. The PNIFECGDB contains two chest channels and 3–4 abdominal channels, taken from a single subject between 21 and 40 weeks of her pregnancy. All recordings were sampled at 1 kHz at a 16-bit resolution. To facilitate the comparison with the state-of-the-art results [7], a total of fourteen recordings (ecga154, 192, 244, 274, 290, 323, 368, 444, 597, 733, 746, 811, 826 and 906) are selected. One minute of signal, 30 seconds after the start of the recording, is extracted for all the available channels of each recording. The true fetal QRS locations were manually annotated and considered to be the reference. The PCDB is the largest publicly available, noninvasive FECG database to date, which consists of 75 one-minute abdominal ECG recordings acquired at a sampling rate of 1 kHz on four channels. The fetal R-peak annotations are provided. Seven recordings (a33, a38, a47, a52, a54, a71 and a74) are excluded, leaving 68 recordings available, due to inaccurate annotations [30].

#### Preprocessing

The prefiltering step for the processing of biological signals is crucial. In the context of fetal QRS and FECG morphology extraction, a low *f*_*l*_ (the cutoff frequency of a high pass filter to remove the baseline wander) should be chosen to preserve the fetal T-waves [9]. Therefore, a bandpass filter with a cutoff frequency of 0.5 Hz (*f*_*b*_) and 100 Hz (*f*_*h*_, the cutoff frequency of a low pass filter to remove the high-frequency content) is applied to the signal. After that, all the signals are normalized using z score normalization [31].

#### Nonlinear adaptive noise cancelling for FECG extraction

Generally, abdominal ECG recordings can be obtained by noninvasive electrodes placed on the maternal abdomen. In the abdominal recording, there are several signal components, and the following equation can be modeled:

AECG(t)=MECG(t)+FECG(t)+n(t),t=1,2,…,T (1)

where *t* is the time; *AECG*(*t*) is the abdominal ECG; *MECG*(*t*) is the maternal ECG in the abdominal ECG; *FECG*(*t*) is the fetal ECG in the abdominal ECG; and *n*(*t*) are various interferences and noise. The abdominal MECG component is formed by the transmission of the maternal ECG from the chest to the abdomen, where the phase and amplitude of the thoracic ECG (TECG) may change significantly. Essentially, this process can be regarded as a complex nonlinear transformation:

MECG(t)=f[TECG(t)] (2)

In Formula (2), the function *f*(∙) represents the nonlinear mapping relationship between the TECG and the abdominal MECG. So long as the best mapping relationship $\hat{f}(∙)$ is available, we can obtain the best estimation of the abdominal MECG. Then, the noisy FECG signal is extracted by subtracting this estimation from the abdominal ECG recording, as shown in Formula (3):

$$\begin{array}{l} \hat{F}=AECG-\hat{M}\\ \;=AECG-\hat{f}(TECG) \end{array}$$ (3)

where $\hat{F}$ is the extracted FECG signal that contains the remaining noise and $\hat{M}$ is the best estimation of the abdominal MECG, which is equivalent to $\hat{f}(TECG)$.

Therefore, the primary task of nonlinear adaptive noise cancelling (ANC) is to obtain the optimal nonlinear transformation function *f*(∙) between the thoracic ECG and the abdominal MECG. In this paper, a deep temporal convolutional network (called TCED-Net) model is constructed as a filter of the ANC architecture to implement the nonlinear mapping of the maternal thoracic ECG to the abdominal MECG, thereby extracting the FECG signal.

#### Model architecture

**Fig 2:**
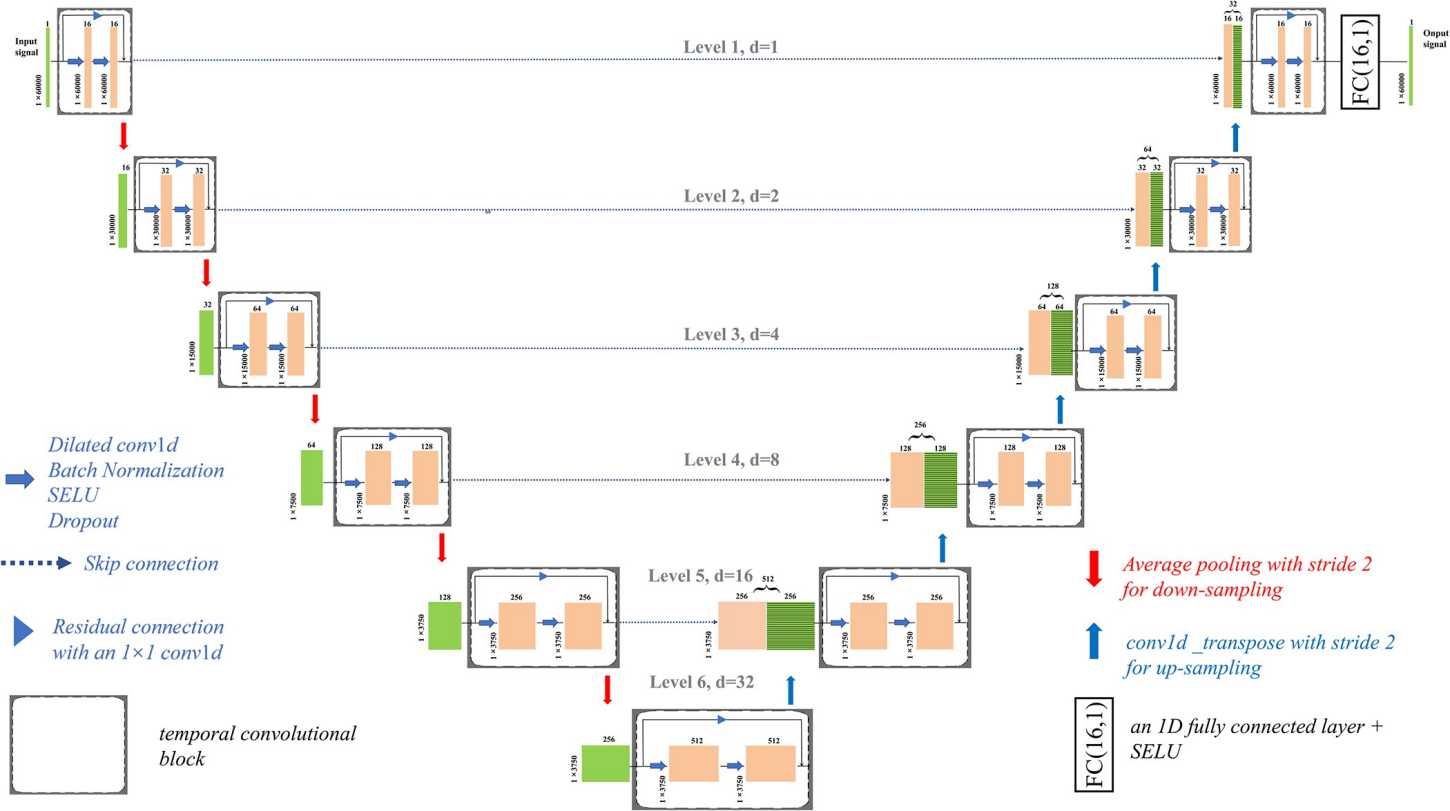
Schematic diagram of our proposed network.

Our network is similar to a generator architecture introduced by Isola et al. [32] in their investigation of conditional adversarial networks to solve image-to-image translations. The difference is that the generator we consider is a U-Net [33] version suitable for the sequence modeling task, corresponding to an encoder-decoder with skip connections between mirrored layers. The network architecture is illustrated in Fig 2; it consists of an encoder (left side) and a decoder (right side). The encoder extracts the salient features of a signal of size 1×60000, preserving the detailed low-frequency structure of the signal. At each extraction step, we double the number of feature channels (the size of the channels is [16, 32, 64, 128, 256, 512], respectively). The temporal convolution block (given in more detail in Fig 4) acts as a feature extractor, in which each orange box corresponds to the multichannel feature maps. The number of channels is denoted on top of the box. Obviously, in a block, convolutions keep the feature maps of the layer the same size as that of the previous one. Each block is followed by an average pooling operation with stride 2 for downsampling. At each downsampling step, we halve the number of feature maps. Each green box represents a set of feature maps cropped from the average pooling, and its x-y-size is provided at the lower left edge of the box. The decoder recovers successive signal details in a bottom-up way from the bottleneck layer of the encoder to produce an output of the same size as the input. At each decoding step, deconvolution is used to upsample the feature maps, which doubles the size of the feature maps and halves the number of feature channels. Each green box with a horizontal line pattern represents a set of feature maps issued from an upsampling layer. Then, we add a skip connection to concatenate the feature maps between the two corresponding encoding and decoding layers, followed by a temporal convolutional block. The skip connection allows the direct shuttling of the signal details from an encoder layer to its corresponding decoder layer, and helps to backpropagate the gradients to the bottom, making training of the end-to-end mapping easier and more effective while the network goes deeper [34, 35]. At the final layer, a 1×1 convolution is used to map the 16-component feature vector to the desired number of channels.

**Fig 3:**
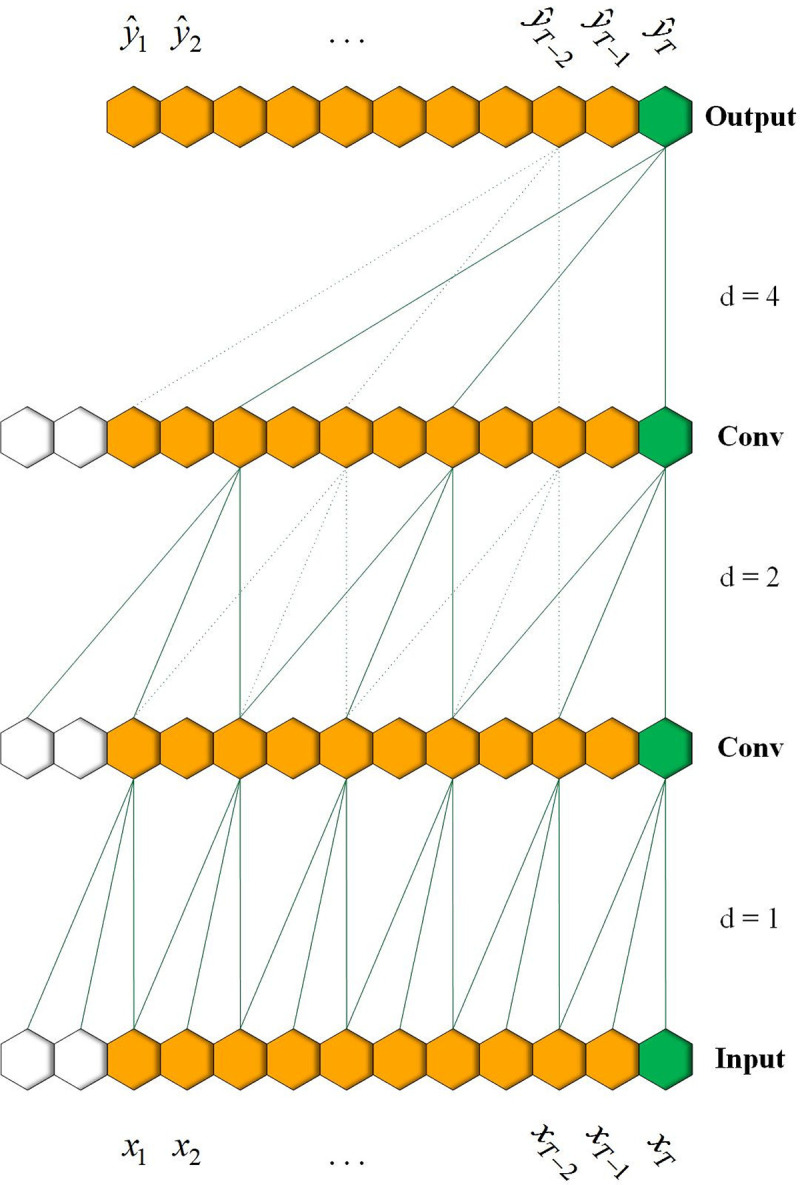
A dilated temporal convolution with factor d = 1, 2, 4 and filter size k = 3.

**Fig 4:**
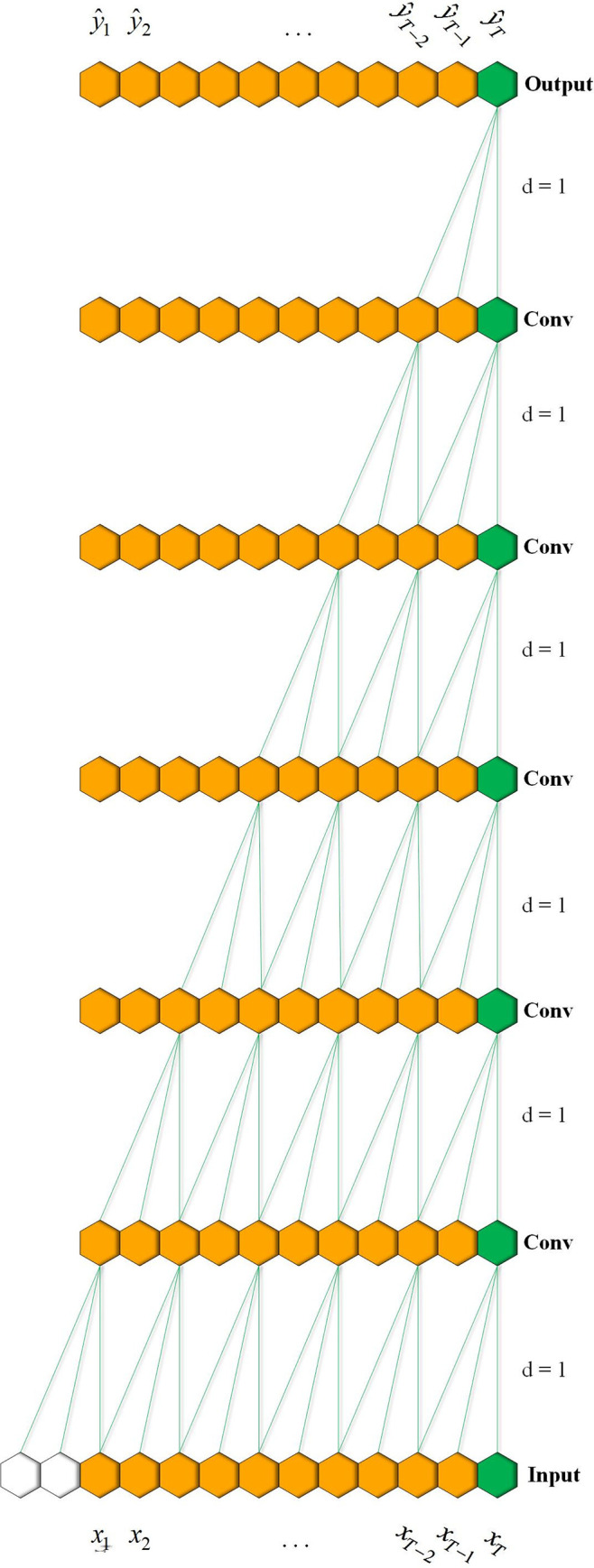
A regular temporal convolution with factor d = 1.

Temporal convolution is used to process the long-term ECG signal in a massive parallel computation, which follows the typical architecture of a convolutional network. For the prediction of the temporal sequence, one distinguishing characteristic is that the convolutions in the architecture are “causal”, where an output at time *t* is involved only with elements from time *t*, and earlier, in the previous layers [36]. However, the length of the history information that a “causal” convolution can look back on is restricted to the depth of the network. To receive a piece of the long history information, we need a deep network or a large receptive field (kernel size), neither of which is particularly feasible. Therefore, following the work of Bai S et al. [36], we use a dilated causal convolution to increase the receptive field. Given a 1-D signal *x*, and a kernel *f*:(0,…,*k*−1}, the output *y* of an element *n* with a dilated convolution is defined as:

$$y(n)=\sum_{i=0}^{k-1}f(i)\cdot x(n-d\cdot i)$$ (4)

where *k* and *d* are the kernel size and the dilation factor, respectively. Note that when *d* is 1, then a dilated convolution is the same as a regular convolution. As shown in Figs 3 and 4, a larger dilation enables an output at the top level to receive a wider range of inputs with fewer layers of the network.

**Fig 5:**
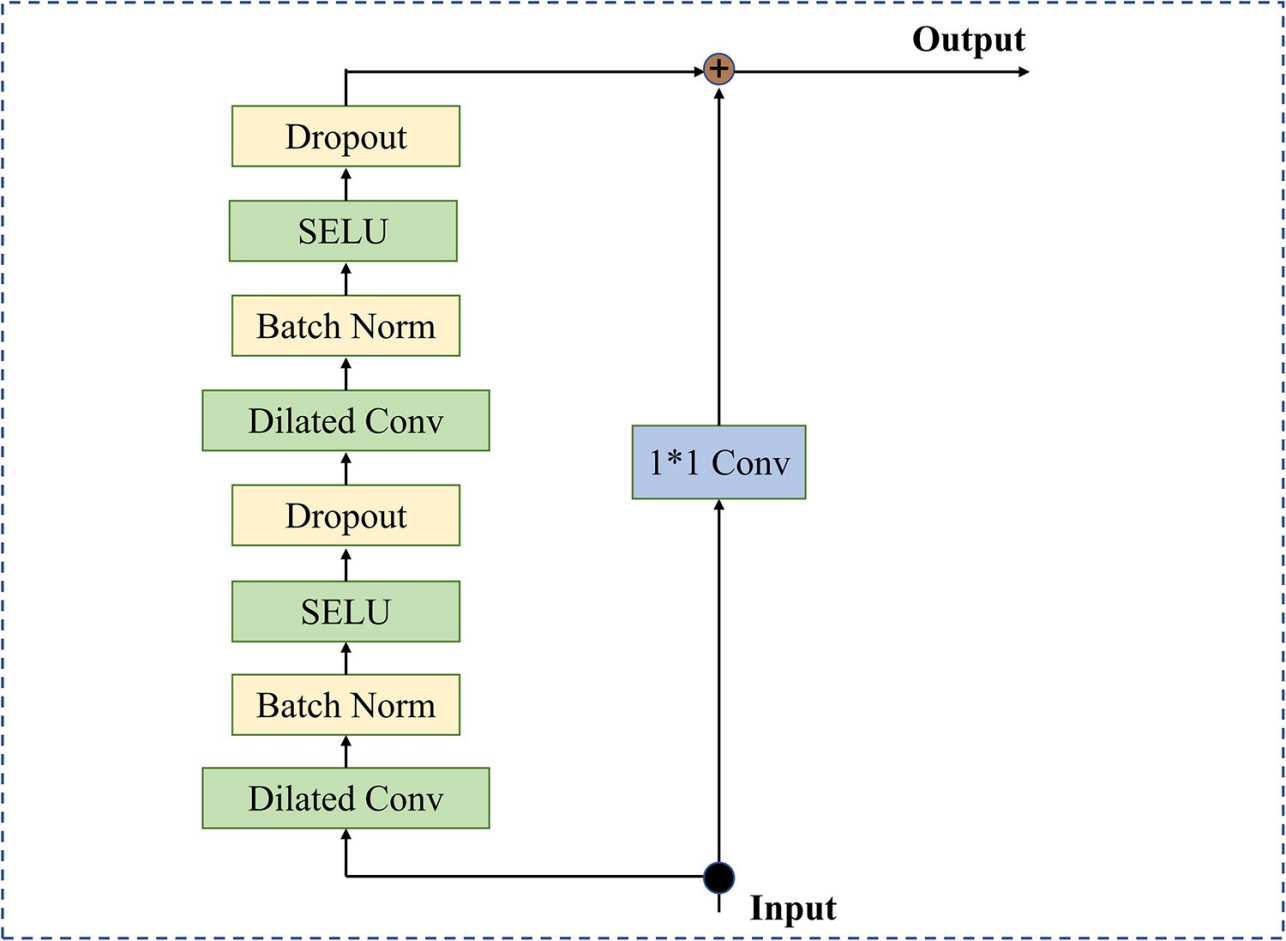
The dilated temporal block used in the TCED-Net of Fig 2.

In our work, the residual block that makes up our network is shown in Fig 5; it consists of two stacked dilated causal convolution layers and nonlinearity layers, for which we use the scaled exponential linear units (SELU) [37]. In addition, regularization operations (batch normalization, spatial dropout) are added to each layer. To achieve stabilization of the deeper or larger network and accelerate the convergence of the model, a residual connection is employed, where a 1×1 convolution ensures that the residual input and output have the same dimension.

### Loss function

For the proposed network, learning the end-to-end mapping, from the mother’s thoracic ECG to abdominal MECG, needs to estimate the weights Θ represented by the convolutional and deconvolutional kernels. This is achieved by minimizing the mean squared loss between the output of the network and the signal to be processed. Specifically, for abdominal MECG elimination, given a collection of N training sample pairs, $D=\{(x_{1},y_{1}),(x_{2},y_{2}),\ldots ,(x_{N},y_{N})\}$, {*x*_1_, *x*_2_,…,*x*_*N*_} are the thoracic ECG sampling points, and {*y*_1_, *y*_2_,…,*y*_*N*_} are the abdominal MECG sampling points as the ground truth. Suppose the output of the network is $\hat{y}=({\hat{y}}_{1},{\hat{y}}_{2},\ldots ,{\hat{y}}_{N})$, i.e.,

$${\hat{y}}_{i}=f(x_{i})$$ (5)

Then, the mean squared error of the network on the training sample pairs is:

$$E(\Theta )=\frac{1}{N}\sum_{i=1}^{N}{\left\{{\hat{y}}_{i}-y_{i}\right\}}^{2}$$ (6)

For the error of Formula (6), given the learning rate η, the parameters of the model are adjusted in the direction of the negative gradient based on the gradient descent strategy. In this paper, we use the PyTorch and Adam optimizers [38] to implement error backpropagation. The detailed hyperparameters of the network are listed in Table 1.

**Table 1:** Hyper-parameters for online optimization of the proposed network.

Hyper-parameter	Considered value	Hyper-parameter	Considered value
Optimizer	Adam	Learning rate *η*	10^−3^
Input signal size	1×60000	Output signal size	1×60000
Feature channels	[16, 32, 64, 128, 256, 512]	Dilated rate	[1, 2, 4, 8, 16, 16]
Stride size of temporal convolution	1	Stride size of down/up sampling	2
Kernel size	5	Padding of the dilated convolution	(kernel size-1)×dilated rate

It is a remarkable fact that the optimal weights Θ in the different channels or recordings are disparate, so the converged models are nonuniversal. In other words, our model utilizes the error between the current thoracic ECG signal and abdominal MECG signal for online optimization and prediction without significant model training or testing.

#### TCED-Net-based ANC framework for abdominal MECG elimination

**Fig 6:**
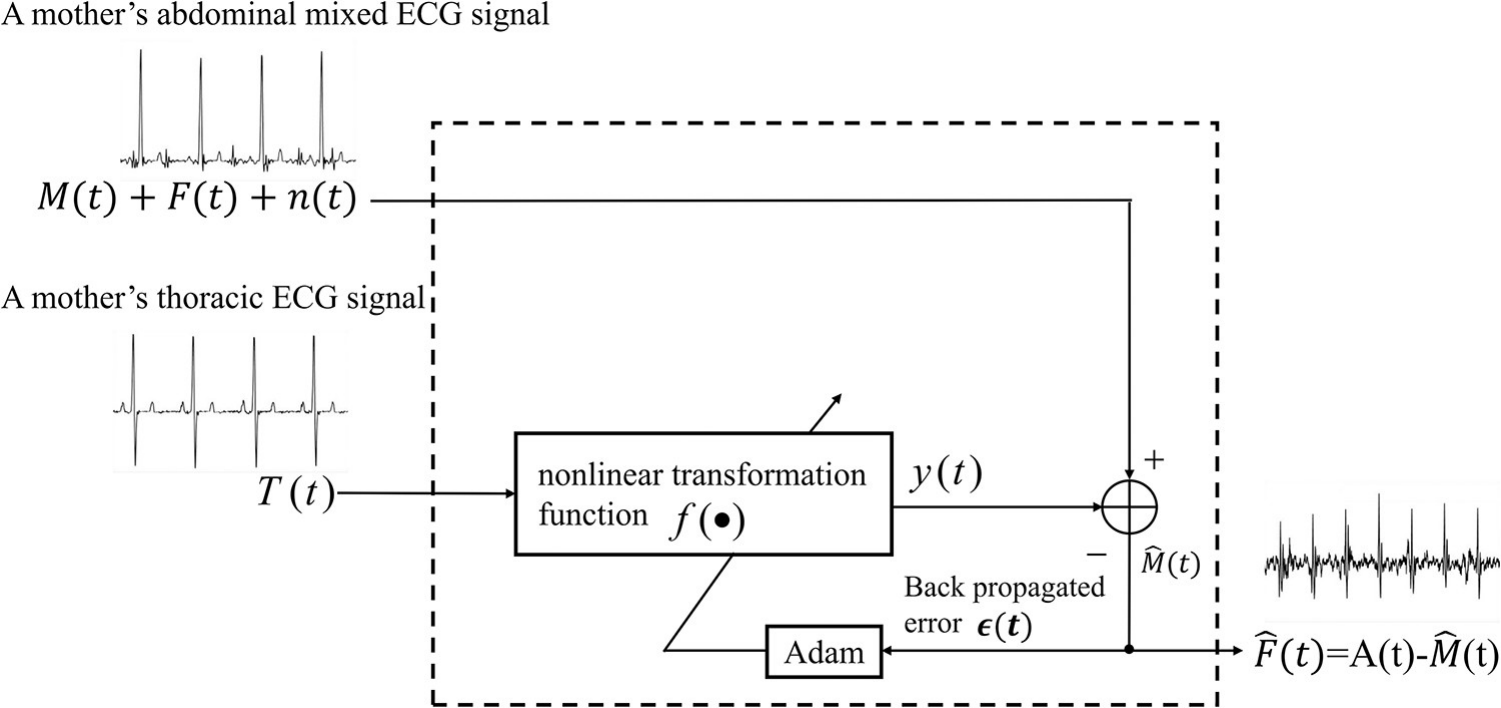
The block diagram of the abdominal MECG elimination using the proposed nonlinear ANC.

In this section, we explain how the proposed network is integrated into the ANC framework, and uses a thoracic ECG to produce the estimation of the abdominal MECG. As mentioned above, the thoracic signal is uncorrelated with the abdominal FECG or noise component but correlated in some unknown way with the abdominal MECG component. Here, the proposed model is essentially a nonlinear transformation function that can be continuously optimized. The block diagram of abdominal MECG elimination using TCED-Net is shown in Fig 6. TCED-Net is denoted as a nonlinear function *f*(∙), and the mother’s thoracic ECG is denoted as *T*. An abdominal ECG to be processed contains the abdominal MECG component *M*, FECG component *F* and noise component *n*. The model transforms the thoracic ECG signal and produces the output *y* = *f*(*T*), and the error is shown in Formula (7).


$$\begin{array}{l} \epsilon =E[{\left(M+F+n-y\right)}^{2}]\\ \;=E[{\left(M-y\right)}^{2}]+2E[\left(M-y)\cdot (F+n\right)]+E[{\left(F+n\right)}^{2}]\\ \;=E[{\left(M-y\right)}^{2}]+2E[\left(M-y)\cdot (F+n\right)]+\\ \;E[F^{2}]+2E[F\cdot n]+E[n^{2}] \end{array}$$ (7)

Since in the abdominal ECG, the fetal ECG, maternal ECG and noise are independent of each other, E[(M−y)⋅(F+n)]≈0,E[F⋅n]≈0. The final error is:

$$\epsilon =E[{\left(M-y\right)}^{2}]+E[F^{2}]+E[n^{2}]$$ (8)

Optimizing the model minimizes the error, we obtain Formula (9).


$$min\;\{\epsilon \}=min\;\{E[{\left(M-y\right)}^{2}]\}+E[F^{2}]+E[n^{2}]$$ (9)

It is obvious that minimizing *ϵ* makes *E*[(*M*−*y*)^2^]→0, equivalent to *y*→*M*. The output *y* is exactly the abdominal MECG component, which is then subtracted from the abdominal ECG. The residual signal comprises the desired FECG and the remaining noise.

#### TCED-Net-based ANC framework for extracted FECG signal denoising

**Fig 7:**
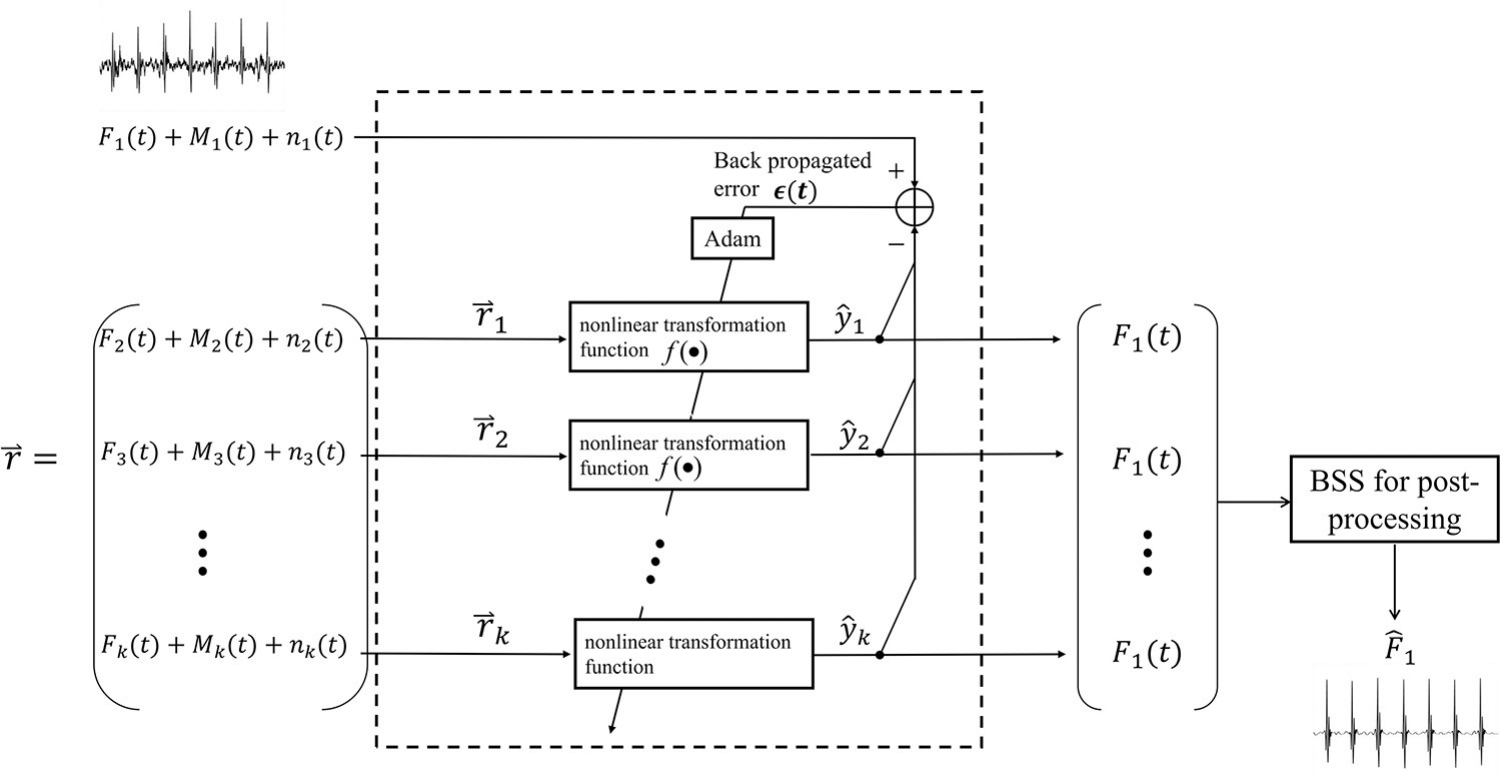
The block diagram of the extracted FECG signal de-noising using the proposed nonlinear ANC. The output is an estimation of the FECG component of channel 1.

The extracted fetal ECG after maternal ECG removal contains a significant amount of residual noise, which may hinder the morphological analysis of the FECG waveform in clinical practice. Typically, the fetal ECG components among the different channels are nonlinearly correlated, whereas some remaining noise components are mostly uncorrelated. In this section, the proposed nonlinear ANC architecture is applied to enhance the quality of the multichannel fetal ECG signals. The block diagram of the *k*-channel extracted FECG signal denoising using TCED-Net is depicted in Fig 7. The channel to be denoised is considered to be the primary channel, and the other channel(s) are considered to be the reference channel(s). We consider that multichannel FECG signals from different acquisition positions can provide more complete information on the fetus, so every channel signal needs to be denoised. The signal components in each channel are shown in Formula (10).

$$\begin{array}{c} x_{1}(t)=F_{1}(t)+M_{1}(t)+n_{1}(t)\\ x_{2}(t)=F_{2}(t)+M_{2}(t)+n_{2}(t)\\ \vdots \\ x_{k}(t)=F_{k}(t)+M_{k}(t)+n_{k}(t) \end{array}$$ (10)

where {*F*_1_(*t*), *F*_2_(*t*),…,*F*_*k*_(*t*)} are the extracted fetal ECG signals, and {*M*_1_(*t*), *M*_2_(*t*),…,*M*_*k*_(*t*)} and {*n*_1_(*t*), *n*_2_(*t*),…,*n*_*k*_(*t*)} are the residual maternal ECG and noises, respectively. In Fig 6, we use channel 1 as the primary channel and the others as reference channels for illustration. ***r*** = [*x*_2_(*t*), *x*_3_(*t*),…,*x*_*k*_(*t*)]^*T*^ is an (*k*−1)×*N* dimensional matrix that is composed of signals of the reference channels. According to the above optimization strategy, given a collection of (k-1) training sample pairs $D=\{(x_{1},r_{1}),(x_{1},r_{2}),\ldots ,(x_{1},r_{k-1})\}$. Suppose the output of the network is:

$${\hat{y}}_{i}=f(r_{i})$$ (11)

where *i*: Ω→{1,…,(*k*−1)} is the index of the reference channels. Then, the mean squared error of the network on the training sample pair (*x*_1_, ***r***_*i*_) is:

$$\begin{array}{l} \epsilon_{i}=\frac{1}{N}\sum_{j=1}^{N}{\left\{x_{1j}-{\hat{y}}_{ij}\right\}}^{2}\\ \;=E[{{(F}_{1}+M_{1}+n_{1}-{\hat{y}}_{i})}^{2}]\\ \;=E[{\left(F_{1}-{\hat{y}}_{i}\right)}^{2}]+2E[\left(F_{1}-{\hat{y}}_{i})*(M_{1}+n_{1}\right)]+E[{\left(M_{1}+n_{1}\right)}^{2}]\\ \;=E[{\left(F_{1}-{\hat{y}}_{i}\right)}^{2}]+2E[\left(F_{1}-{\hat{y}}_{i})*(M_{1}+n_{1}\right)]+E[{M_{1}}^{2}]+2E[M_{1}*n_{1}]+E[{n_{1}}^{2}] \end{array}$$ (12)

As mentioned above, the fetal ECG components among the different channels are nonlinearly correlated, whereas some remaining maternal ECG and noise components are mostly uncorrelated. Therefore, we know that $E[\left(F_{1}-{\hat{y}}_{i})*(M_{1}+n_{1}\right)]\approx 0,E[M_{1}*n_{1}]\approx 0$. The final error is:

$$\epsilon_{i}=E[{\left(F_{1}-{\hat{y}}_{i}\right)}^{2}]+E[{M_{1}}^{2}]+E[{n_{1}}^{2}]$$ (13)

Similar to the maternal ECG estimation scheme, optimizing the model minimizes the error, and we obtain Formula (14).


$$min\;\{\epsilon_{i}\}=min\;\{E[{\left(F_{1}-{\hat{y}}_{i}\right)}^{2}]\}+E[{M_{1}}^{2}]+E[{n_{1}}^{2}]$$ (14)

Obviously, minimizing *ϵ*_*i*_ would make $E[{\left(F_{1}-{\hat{y}}_{i}\right)}^{2}]\to 0$, equivalent to ${\hat{y}}_{i}\to F_{1}$. The output ${\hat{y}}_{i}$ of the network is exactly the desired clean FECG component of channel 1. Compared to the MECG estimation, the main difference of the FECG denoising is that the target signal is the output ${\hat{y}}_{i}$ of the network, instead of the error *ϵ*_*i*_.

As explained above, *i*: Ω→{1,…,(*k*−1)} is the index of the reference channels. Going through a loop that iterates over *i*, we obtain a set of fetal ECG signals that are approximate to the FECG component in channel 1.

### Postprocessing

In this section, a BSS step is used to process the set of fetal ECG signals and to finally extract the fetal ECG of channel 1. BSS methods are the most commonly used techniques for multichannel signal separation. In the literature [29], the BSS performance was assessed through three different algorithmic implementations (PCA, JADE and fast-ICA). Finally, the JADE algorithm achieved the best result using eight channels. For this reason, JADE is selected as the postprocessing algorithm added after the proposed framework.

### Statistical assessment

The FECGSYNDB provides the fetal QRS complex locations, and the “true” fetal ECG waveforms, prior to mixing with MECG and the other noise sources, which enables the assessment of both the fetal QRS detection accuracy and the ability to conserve fetal ECG morphological features. For PNIFECGDB and PCDB, only the locations of the fetal QRS complexes are available. Therefore, we used sensitivity (SE), positive predictive value (PPV) and the *F*_1_-score to evaluate the performance of the proposed method on real clinical data.

$$SE=\frac{TP}{TP+FN}\cdot 100(\%)$$ (15)

$$PPV=\frac{TP}{TP+FP}\cdot 100(\%)$$ (16)

$$F_{1}=\frac{2\cdot TP}{2\cdot TP+FP+FN}\cdot 100(\%)$$ (17)

where TP denotes the number of correctly identified R peaks within 50 ms of the reference annotations [39]. FN indicates the number of missed R peak detections. FP is the number of falsely detected nonexistent peaks.

Accurate fetal QRS detection enables accurate FHR tracings. However, accurate fetal QRS detection does not necessarily imply that the morphology of the extracted fetal ECG is well preserved. In terms of assessing how accurately the morphological information of the extracted FECG signal can be preserved, mean squared error (MSE), signal-to-noise ratio (SNR) and the correlation coefficient (R) are used as the performance indices.

$$MSE=\frac{1}{N}\sum_{i=1}^{N}{\left(x_{i}-\hat{x_{i}}\right)}^{2}$$ (18)

$$SNR=10log10\frac{\sum_{i=1}^{N}{\left(x_{i}\right)}^{2}}{\sum_{i=1}^{N}{\left(x_{i}-\hat{x_{i}}\right)}^{2}}$$ (19)

$$R=\frac{\sum_{i=1}^{N}\left(x_{i}-{\bar{x}}_{i})(\hat{x_{i}}-{\bar{\hat{x}}}_{i}\right)}{\sqrt{\sum_{i=1}^{N}{\left(x_{i}-{\bar{x}}_{i}\right)}^{2}}\sqrt{\sum_{i=1}^{N}{\left(x_{i}-{\bar{\hat{x}}}_{i}\right)}^{2}}}$$ (20)

where ${\hat{x}}_{i}$ is the fetal ECG extracted from the abdominal ECG, and *x*_*i*_ is the synthetic fetal ECG as the ground truth. ${\bar{x}}_{i}$ and ${\bar{\hat{x}}}_{i}$ indicate the means of the two signals. *N* is the number of samples in the signal. Generally, a larger SNR and R, or a smaller MSE, indicates a higher accuracy of an algorithm.

## Results

In this work, qualitative and quantitative analyses (e.g., the visualization of FECG morphology and the detection of fetal QRS complexes) are conducted to evaluate the performance of the proposed framework for FECG extraction. For the task of maternal ECG estimation, the thoracic ECG signal usually has a high SNR, and the model is optimized easily as well, which may increase the risk of overfitting. Therefore, we set the number of training epochs to 100. In contrast, for the FECG denoising, the extracted fetal ECG in each channel contains a significant amount of remaining noise, which makes the model convergence difficult. The corresponding training epochs are set to 200. Before applying the proposed method and its counterparts, we preprocess all the recordings.

### Experimental results on the simulated data from the FECGSYNDB

The proposed method is compared with different state-of-the-art techniques, including LMS, RLS, ES-RNN, TS and EKF. We use synthesized ECG signals to investigate the accuracy of the extracted fetal ECG morphology, as well as for the fetal QRS detection accuracy. Referring to the literature [29], a combination of four abdominal channels (1, 11, 22 and 32) and the first thoracic channel are used, where the noise level is 0 dB.

**Fig 8:**
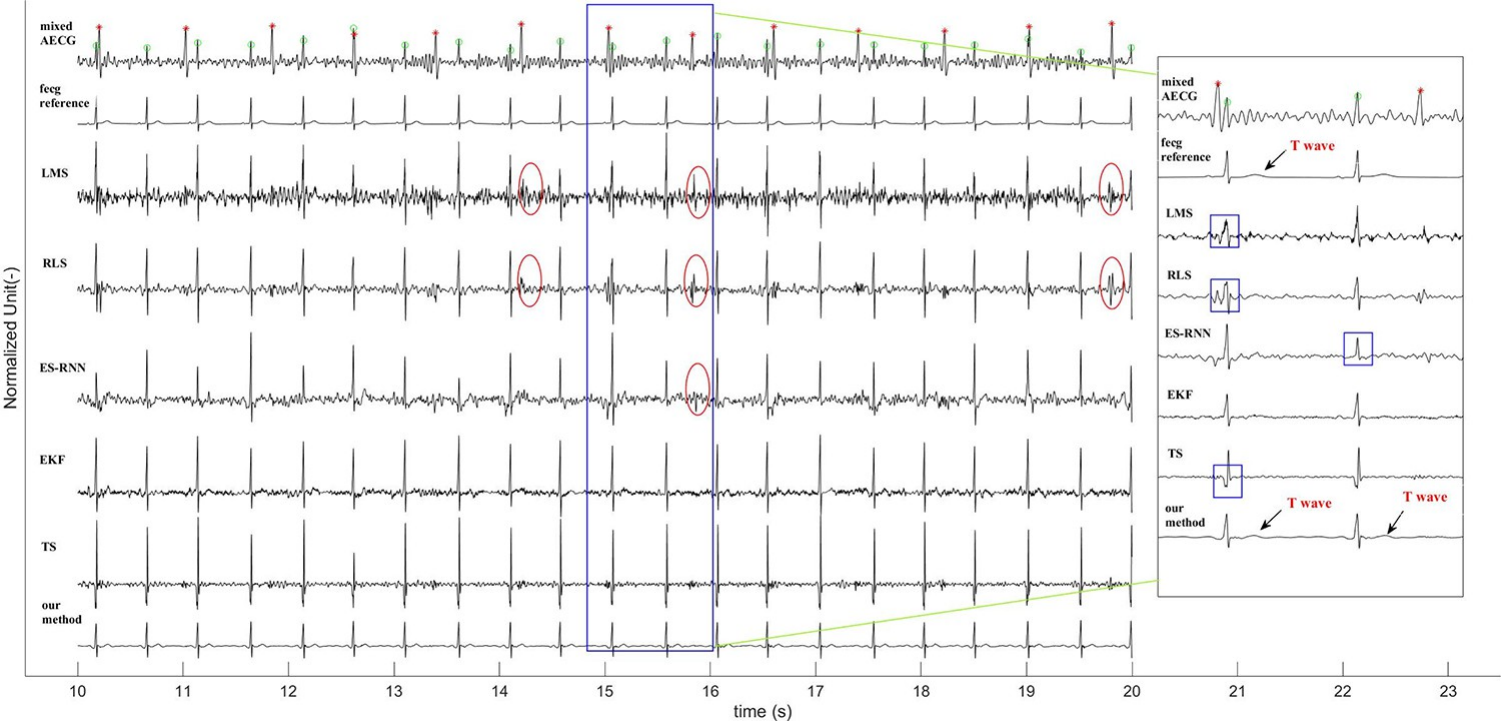
The visual comparison of extracted FECG signals among different methods where the noise level is 0 dB. And all signals are normalized for better observation.

To intuitively reflect the capabilities of the proposed method, the visualization of an example of the extracted FECG is depicted in Fig 8. We can see that our framework achieves a notable performance against the existing techniques in preserving the morphological features of the FECG signal extracted from the AECG recording. Specifically, the MECG component suppression effect of the existing methods is not ideal, and the residual MECG components remain (see the red ellipse box in Fig 8). It should be emphasized, that in the case of overlapping of the fetal QRS and maternal QRS, the fetal QRS of the existing methods are significantly distorted (see the blue rectangular box in the subfigure of Fig 8). The above phenomenon will severely disrupt the subsequent FQRS detection and morphology analyses of the FECG waves. We notice that although the morphological features of the FECG extracted by the EKF are obvious, as shown in the subfigure, its weak features, such as the T waves, are lost. From the last row of Fig 8, we can see that the morphology of the FECG extracted by our method is clean without obvious MECG residuals. Another aspect is that the FECG’s T waveforms are preserved, making evaluating fetal abnormalities through QT interval measurements possible.

The statistical results (average *F*_1_-score, MSE, average SNR and average R) of the methods under the 0 dB noise level are tabulated in Table 2. These statistical indices are computed by the extracted locations of the R-peaks and wave morphology against the reference annotations. The results shown in Table 2 indicate that our method is reliable on its own. In all cases, it achieves the best performance against the other state-of-the-art techniques.

**Table 2:** The statistical results of different methods on the FECGSYNDB.

Methods	Noise level (dB)	*F*_1_-score	MSE	SNR	R
LMS	0	86.21	0.68	1.66	0.52
TS	0	88.31	0.40	3.96	0.82
RLS	0	90.20	0.48	3.18	0.76
EKF	0	94.84	0.38	4.26	0.86
ES-RNN	0	92.77	0.45	3.48	0.80
Our method	0	98.89	0.16	7.94	0.95

### Experimental results on clinical ECG data from the PNIFECGDB

**Fig 9:**
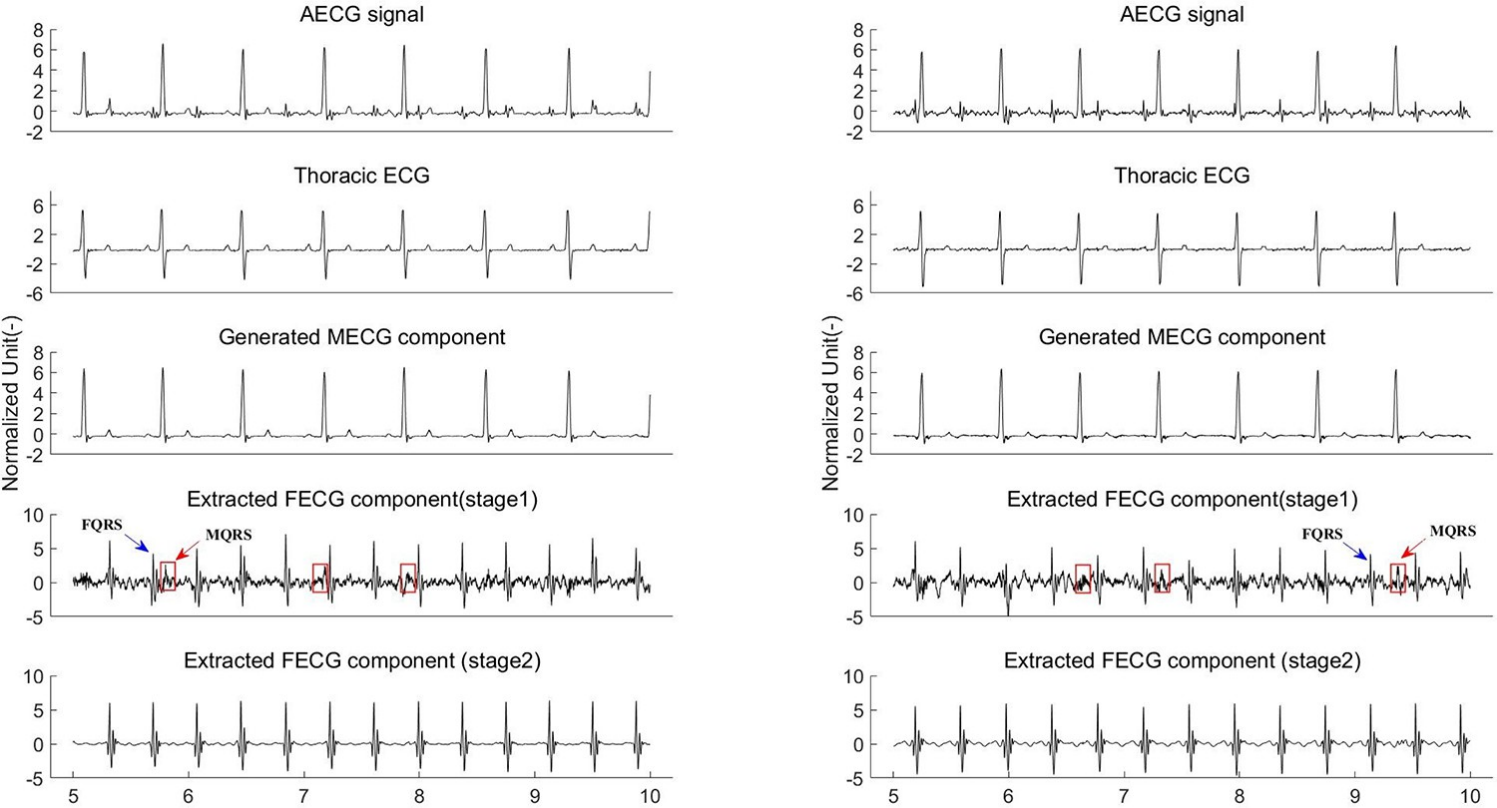
FECG extraction using the proposed framework on PNIFECGDB data. The left one is the extracted FECG and fetal HR signals on channel-1 of the recording “244”. The right one is the result on channel-1 of “811”. All signals are normalized for better observation.

Following the suggestion of [7], a total of fourteen recordings are selected. For each recording, the first three abdominal channels and the first thoracic channel are kept to make fair comparisons among the different methods. Since a reference fetal ECG is not available, we conducted a quantitative analysis of the fetal QRS detection accuracy. Examples of two abdominal ECG signals of recordings “ecgca 244” and “ecgca 811” and a fetal ECG extracted using the proposed framework are shown in Fig 9. The original signal is in the first row, where the fetal ECG component is very weak compared to the maternal ECG component. Typically, the ECG characteristics collected from the maternal body are not invariable and often change with the changes in electrode placements. As shown in the second row, we show a thoracic ECG signal that is recorded at the maternal chest. Obviously, there is a loose similarity between the thoracic ECG and the AECG recording. The third row shows the maternal ECG component estimated by our network using the reference ECG signal, which is morphologically similar to the abdominal maternal ECG waveform. The fetal ECG signal is extracted so long as we subtract the estimated maternal ECG component from the abdominal ECG, as shown in the fourth row. However, there are obvious maternal ECG components remaining in the extracted fetal ECG signal (see the red ellipse box in Fig 9), which may be mistakenly detected as fetal QRS features. Thus, denoising the extracted noisy fetal ECG signal to enhance its quality becomes of paramount importance. We obtain a fetal ECG signal with clean morphology according to the above scheme for fetal ECG denoising (see the fifth row of Fig 9). It should be emphasized, that although the amplitude of the fetal ECG is much lower than that of the maternal ECG, the suggested method still produces an ECG signal with relatively clean morphology, especially for the T wave and QRS complex.

**Fig 10:**
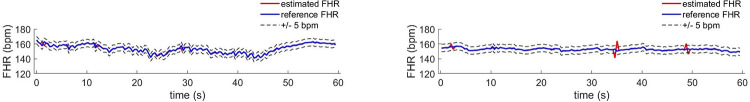
Smooth fetal HR traces extracted by our method.

In Table 3, we report the average *F*_1_-score of our method and of other approaches. The results shown in Table 3 indicate that our method outperforms most existing techniques and extracts more fetal QRS features (*F*_1_-score = 98.94%). It is worth noting that accurate beat-to-beat detection of fetal R peaks is needed for computing the fetal heart rate (HR) signal. Smooth fetal HR traces extracted by our method are shown in Fig 10.

**Table 3:** The statistical results of different methods on the PNIFECGDB.

Methods	SE (%)	PPV (%)	*F*_1_-score (%)	Number of channels	Matching window length (ms)
RLS	96.42	95.86	96.14		
TSpca [9]	94.41	95.69	95.05		
ES-RNN [7]	96.99	97.16	97.07	The first three abdominal channels and first thoracic channel	50
FLANN [40]	97.08	97.29	97.19		
GFLANN [41]	98.03	98.92	98.47		
Our method	99.02	98.86	98.94		

### Experimental results on clinical ECG data from the PCDB

**Fig 11:**
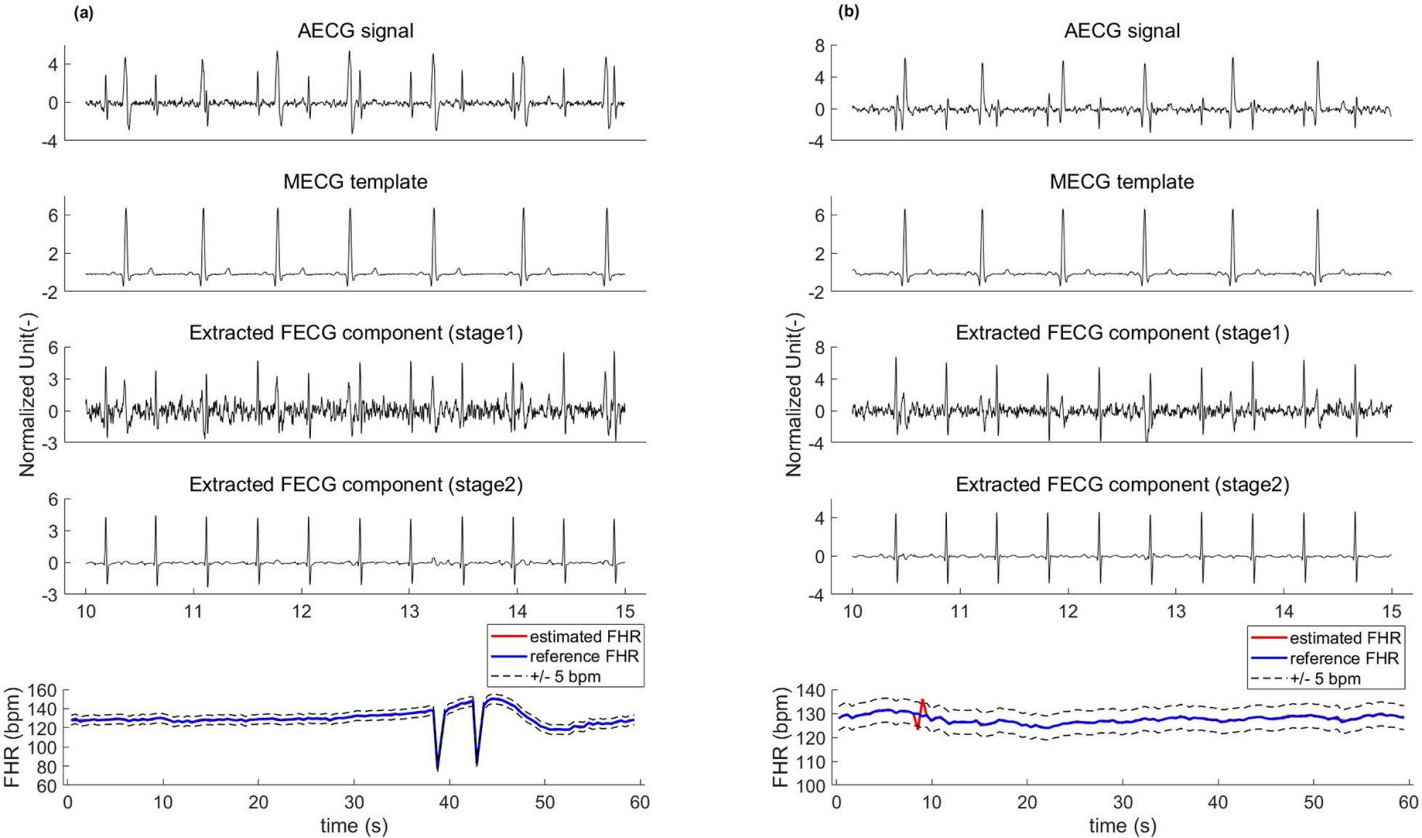
FECG extraction using the proposed framework on PCDB data. (a) is a sample from “a04” channel-1, (b) a sample from “a08” channel-1.

**Fig 12:**
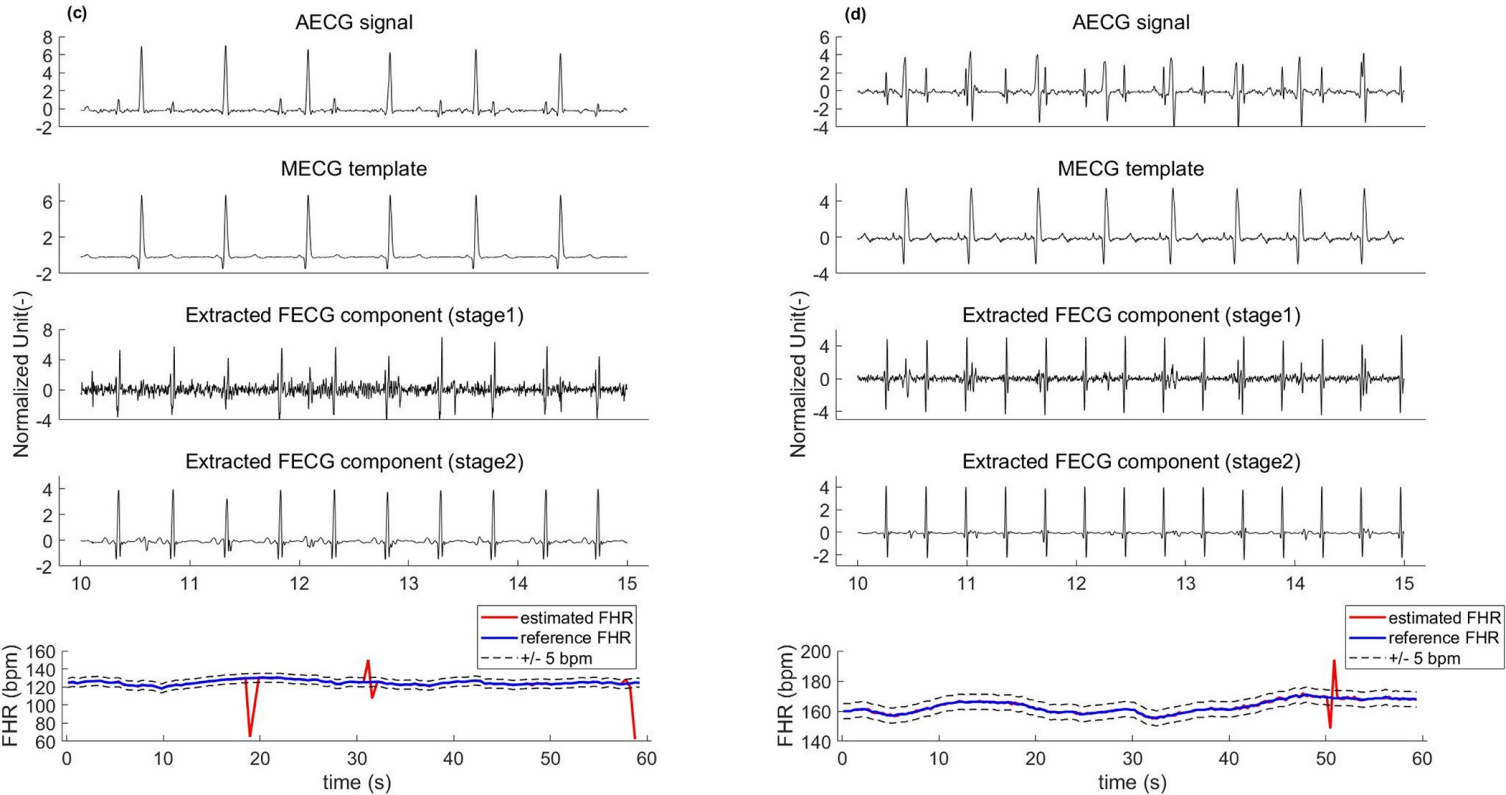
FECG extraction using the proposed framework on PCDB data. (c) a sample from “a25” channel-1 and (d) a sample from “a44” channel-1. All signals are normalized for better observation.

The above work needs an additional mother’s thoracic ECG as a reference to achieve reasonable maternal ECG removal from the abdominal ECG, which is inconvenient in practical clinical applications. This section investigates the performance of the proposed framework on the PCDB in the absence of a thoracic ECG reference. The PCDB represents the largest publicly available fetal ECG dataset to date where each recording contains four abdominal channels without any reference thoracic ECGs. Specifically, we build an average maternal ECG cycle (the synthetic thoracic ECG reference) by detecting the locations of the maternal R peaks from the abdominal signal as the replacement of a real thoracic ECG signal. Figs 11 and 12 show the performance of the proposed method on several samples from PCDB. In each subfigure, the original signal and the maternal ECG template are in the first and second rows, respectively. It can be seen that the synthetic reference does not correctly represent the maternal ECG waveform of the mother’s abdomen. After subtracting the maternal ECG component that is estimated by our network using the synthetic thoracic ECG reference from the abdominal ECG, we obtain the noisy fetal ECG signal, as shown in the third row. The fourth row shows that the proposed fetal ECG denoising scheme effectively suppresses the residual maternal ECG and noise, extracting the FECG signal with relatively clean morphological features. However, because the artificial maternal ECG differs from the real thoracic ECG, the waveform of the extracted fetal ECG is distorted to some degree, and some small features (e.g., the P and T waves) are lost.

Table 4 shows a performance comparison among the different techniques that do not require maternal ECG reference on the PCDB. As shown in Table 4, our framework outperforms the other state-of-the-art methods in terms of fetal QRS detection accuracy, achieving SE, PPV and *F*_1_-scores of 98.61%, 98.63% and 98.62%, respectively. The corresponding fetal heart rate (HR) signal computed by our method is shown in the last row of Figs 11 and 12 (the red trace is the estimated fetal HR signal, and the blue trace is the reference fetal HR signal).

**Table 4:** The statistical results of different methods on the PCDB.

Methods	SE (%)	PPV (%)	*F*_1_-score (%)	Matching window length (ms)
Cerutti [29]	86.39	87.08	86.66	
TSpca [9]	89.93	91.78	90.82	
STFT-GAN [42]	92.37	93.69	93.02	
RCED-Net [21]	92.60	94.68	93.62	
FUSE [43]	95.98	95.15	95.50	50
AECG-DecompNet [6]	93.52	97.41	95.43	
1-D Octave Convolution [3]	90.32	91.82	91.06	
Ensemble Kalman Filter [44]	96.91	97.59	97.25	
Convolutional Neural Network [23]	89.06	92.77	90.88	
Our method	98.61	98.63	98.62	

## Discussion

Although the accurate detection of fetal QRS complexes is necessary for computing the fetal heart rate (HR) trace, we believe that the main space for innovation in the field of fetal ECG is in designing algorithms for morphological analysis of the waveform. Extracting morphological features of the fetal ECG signal allows for a diagnosis of the well-being of the fetus. Fetal acidosis is known to affect ECG morphology, while asphyxia of the fetus is thought to be associated with changes in the P wave, PQ interval and ST segment. In this study, we propose a framework comprised of two networks to decompose both the maternal ECG and fetal ECG signals from the mother’s abdominal ECG recording. As opposed to the existing techniques being restricted to the extraction accuracy of the fetal QRS complexes, the main goal of this research is to extract a fetal ECG signal with clean morphological waveforms.

With the help of the mother’s thoracic ECG, using the first network to estimate the maternal ECG, and subtracting this from the abdominal ECG recording, is similar to the adaptive noise cancelling (ANC) technique. However, in a classical linear ANC, to achieve a reasonable MECG removal, the reference signal (thoracic ECG) should be morphologically similar to the abdominal maternal ECG component. It should be noted, that in most cases, the ECG characteristics collected from the body of a pregnant woman are not invariable, and often change with the change in electrode placements. Thus, considering that the relationship between the thoracic ECG and the abdominal maternal ECG is nonlinear, a deep temporal convolution model is proposed as a nonlinear filter of the ANC architecture to map the maternal ECG from the mother’s chest to the abdomen. After the maternal ECG is removed, the proposed adaptive nonlinear filter is also applied to enhance the quality of the extracted multichannel fetal ECG signals that still contain a substantial amount of noise. Concretely, the channel to be denoised, pairing with the other channels (reference channels), is sent into the nonlinear ANC to produce a sequence equivalent to the fetal ECG component of the channel to be denoised. Going through a loop that iterates over a number of reference channels, we obtain a set of fetal ECG signals that are approximate to the fetal ECG component in the channel to be denoised. Then, JADE (one of the blind source separation algorithms) as the postprocessing step is applied to the collection, and finally produces a clean FECG signal.

The key to the performance of the proposed framework is that the network can adaptively learn the end-to-end mapping from the mother’s thoracic ECG to the abdominal ECG. The experimental results on several datasets thus far are promising. We have verified the feasibility of our method using synthetic ECG data (FECGSYNDB) and clinical ECG data (PNIFECGDB), which can extract the fetal ECG signal with morphological features from the abdominal ECG recording using the thoracic ECG as a reference (Figs 8 and 9 and Tables 2 and 3). Considering that it is difficult to acquire the mother’s thoracic ECG reference in clinical practice, we then generate an artificial maternal ECG as a reference of the clinical ECG data (PCDB) to further investigate the reliability of our algorithm. Figs 11 and 12 indicate that despite the significant difference between the synthetic ECG reference and the abdominal maternal ECG, the proposed model can effectively suppress the maternal ECG component and extract a relatively clean fetal ECG signal. In Table 4, we compare our method with existing related works that do not require a maternal ECG reference. The results prove that without a real thoracic ECG as a reference, our method is robust and has superior performance over the existing related works.

### Contributions and advantages

The contribution of this research focuses on the processing of the mother’s abdominal ECG recordings and the removal of the abdominal maternal ECG. However, the improved nonlinear filter is found to be effective in denoising the extracted fetal ECG signal. After filtering, the morphology of the fetal ECG waveform is retained to the extent that even the small signal waves can be visually distinguished.

Compared with the deep learning-based methods that extract the fetal ECG directly from the abdominal ECG, the proposed framework consists of two extraction networks (one for MECG elimination and the other for FECG denoising) and a blind source separation (JADE), which is capable of extracting the fetal ECG with high accuracy in QRS detection while better preserving most of the morphological features. In addition, different from the offline optimization of the existing extraction models, the adaptive filtering scheme is applied to adjust the model’s parameters autonomously to remove the undesired components (e.g., maternal ECG and noise) from the abdominal ECG signal. Specifically, in the ANC architecture, our model utilizes the error between the current reference signal and the input signal for online optimization and prediction. As a result, our model requires no significant model training or testing while achieving a higher or comparable performance to the state-of-the-art methods. Finally, in the case of fewer layers of the network, the small kernel of a regular CNN cannot perceive the long quantity of historical information of the ECG signal to make the prediction. The dilated causal convolution increases the receptive field to capture the long-term temporal dynamics of the long-term ECG signal, which corresponds to the long-term memory of the LSTM. Another benefit of the dilated convolution operation is the reduction in computational cost and memory footprint, enabling implementations on resource-constrained devices. In contrast with the recurrent neural network (RNN), the convolution-based model implements the massively parallel computing of the long-term ECG signal via GPU computation and, as such, is rather generally practical.

### Limitations

Our extraction framework has some limitations. First, to achieve reasonable maternal ECG removals, the mother’s thoracic ECG reference should be acquired synchronously with the abdominal ECG recording, which is inconvenient in practical clinical applications. We built an artificial thoracic ECG reference by detecting the locations of the maternal R peaks from the abdominal signal as a replacement in the absence of the thoracic reference ECG signals. However, this operation involves estimating QRS complex waveforms of the maternal ECG from the abdominal ECG, which may be a challenging problem in situations where the fetal ECG and maternal ECG waveforms temporally overlap. Second, the number of network iterations (“epoch”) is an empirical value that can only be adjusted according to the experimental results. To avoid overfitting or underfitting of the network, the value of “epoch” should not be too large or small.

## Conclusion

In this paper, a framework consisting of two-stage temporal convolutional encoder-decoder networks, and a blind source separation (JADE), is investigated to eliminate the maternal ECG, and to extract a clean fetal ECG from the abdominal ECG. As opposed to the existing algorithms being restricted to fetal QRS complex detections, the extraction goal of our method is to preserve the morphological features of the fetal ECG waveform. The statistical and visual results show that our method has the potential to implement the screening of fetal well-being through an analysis of both the FHR and fetal ECG waveform morphological features. Future work could focus on performing extractions of the relevant morphological features such as the PR intervals, QT intervals, and ST segments to advance the research toward machine learning for automated diagnosis and classification of fetal health.
